# Availability, price, and affordability of medicines used for the management of Covid-19 in health facilities of Dessie town WHO/HAI survey

**DOI:** 10.1371/journal.pone.0279465

**Published:** 2022-12-21

**Authors:** Solomon Ahmed Mohammed, Tessema Tsehay, Abebe Getie Faris, Getnet Mengstu

**Affiliations:** Department of Pharmacy, College of Health Sciences, Wollo University, Dessie, Ethiopia; University of Mississippi Medical Center, UNITED STATES

## Abstract

**Background:**

The rapidly spreading nature of Covid-19 virus associated with its high mortality and mortality rate is triggering an unprecedented public health crisis. The study assessed the availability, price, and affordability of medicines used in the management of Covid-19 in health facilities of Dessie town.

**Methods:**

A retrospective cross-sectional study design was employed in the health facilities of Dessie town from September 1 to September 20, 2021. Data was collected using a standard checklist adopted from the Logistics Indicator Assessment Tool and WHO/HAI. WHO/HAI methodology was applied to select the surveyed health facilities (30) and medicines (44). The daily wage of the lowest-paid unskilled government worker is used to estimate affordability.

**Results:**

Fifteen and five medicines were not found at all public and private health facilities, respectively. The originator brand (OB) and lowest price generic (LPG) availability in private health facilities was 2.03% and 51.33%, respectively. In the public sector, the availability of OB and LPG was 0% and 34.44%, respectively. In public and private health facilities, the mean number of stock-outs was 2.25 and 2.91, and the mean number of stock-out days was 177.83 and 106.16 days, respectively. Eight and one LPG medicines were out of stock in public and private health facilities, respectively. Eight (33.33%) and 6 (28.57%) had higher prices than international prices in private and public health facilities, respectively. The median price ratio in public and private health facilities ranged from 0.02 and 3.05 and 0.04 to 2.70, respectively. Eighty percent of the products were unaffordable in both sectors.

**Conclusions:**

The availability of medicines was low. One-third of the medicines had higher prices than international prices. Eighty percent of the products were unaffordable. The regular supply of these medicines is crucial for better management of the disease.

## Introduction

Coronavirus disease 2019 (COVID-19) is an illness caused by severe acute respiratory syndrome coronavirus 2(SARS-CoV-2). It was turned into a global pandemic in 2020 after the declaration made by World Health Organization (WHO) [1]. Ethiopia confirmed the first COVID-19 case on March 13, 2020 [2]. COVID-19 is still a main public health problem. The rapidly spreading nature of the virus associated with its high mortality and mortality rate is triggering an unprecedented public health crisis [3].

So far, there are no antiviral drugs or vaccines used to treat COVID-19 [1]. As the outbreak was first identified in China, the country was severely affected by the reduced flow of the medical supply chain due to the lockdown [4]. To tackle this problem, the Food and Drug Administration (FDA) and the American Society of Health-System Pharmacists (ASHP) developed a drug shortage list that included the potential COVID-19 treatments and related infections [5].

According to WHO, one-third of the world’s population lacks access to essential medicine [6, 7]. The magnitudes of the problem worsen in developing countries. Various factors contributed to the inaccessibility of medicines such as price, rational use, availability of finance, and supply chain systems [8]. Globally, various studies reported confirmed shortages elsewhere in the world. The shortage of essential medicines was reported in Saudi Arabia [9], Bangladesh [10], and Nigeria [11]. In Ethiopia, there are concerns about the regular supply of drugs [2]. The shortage of essential medicines was reported and it is particularly serious as COVID-19 has no clear treatment and these medicines are used to treat complications and autoimmune diseases [12]. Personal protective equipment was also faced with stock outs. The shortage has been attributed to increased demand [13].

Patents have the right to access medication. Accessibility of medicines is the steady availability and affordability of medicines in both public and private health facilities [14]. However, COVID-19 occurrence affected the availability of suppliers and the competitiveness of the market especially in developing countries [15]. Strengthening the prevention program to halt the spread of disease and the selection of a list of priority medicines to have a unified and evidence-based treatment plan should be considered to optimize the use of medicines [3].

Nearly 30% of the African and 9.1% of the sub-Saharan African population will fall into extreme poverty and will be unable to recover from the economic crises [16]. COVID-19 is increasing the demand for prescriptions [17]. Therefore, a significant number of patients are expected to lose their already fragile ability to buy medicines [16]. To mitigate the risks of inaccessibility of essential medicines due to the current crisis, governments should implement policies to protect patients that are socially disadvantaged and underserved groups [18]. Moreover, access to medicines can be increased by counteracting any barriers that may hinder the affordability of medicines [8].

Findings of researches conducted in Bangladesh [10], India [19], Nigeria [11], and Rwanda [20] revealed that the price of essential medicines was increased. In Rwanda, despite essential medicines prices in the private sector being less than the international purchase prices, the price was doubled [20]. This made an increased demand for imported medicines and ultimately caused the prices of medicines to accelerate [21].

The availability, price, and affordability of medicines to patients are crucial. However, the COVID-19 pandemic affected the medical supply chain and created serious challenges to the quality of health care. Therefore, this study aimed to assess the availability, price, and affordability of medicines used to manage COVID-19.

## Methods

### Study area and period

The study was conducted from September 1 to September 20, 2021, in the health facilities of Dessie town, North-East Ethiopia. Dessie is one of the towns in the Amhara region preferred for medical tourism. In the town, there are 8 public health centers, one comprehensive hospital, one district hospital, 7 private hospitals, and 30 pharmacies. Public health facilities are managed by the local government and procurement of medicines was done independently by the Ethiopian Pharmaceutical supply agency (EPSA). Private pharmacies and private hospitals independently procure their drugs from wholesalers. The list of health institutions was obtained from the town health administration.

### Study design

A retrospective cross-sectional study was conducted to determine the availability, price, and affordability of medicines used for the management of Covid-19 in the health facilities of Dessie town.

### Study population and source population

The source populations for this study were all medicines available in health facilities. The study populations were medicines used for the management of Covid-19 in the health facilities.

### Inclusion and exclusion criteria

All medicines used for the management of Covid-19 in the health facilities of Dessie town were included. Medicines that are not registered in Ethiopia and medicines that had incomplete records were excluded. We exclude drug stores as only a few medications were handled by them.

### Sample size determination

#### Selection of healthcare facilities

According to WHO/HAI, a major center will be selected and an additional five survey areas will be selected randomly from all the administrative areas that can be accessed within one day of travel from the major center through a bus. For each selected survey area, five public health facilities and four public sector medicine outlets will be selected randomly within three hours’ travel from the main hospital. In areas that have less number of health facilities, medicine outlets that are closest to each public medicine outlet will be selected [8]. Accordingly, 30 (20 private and 10 public) medicine outlets were selected for the study based on the WHO/HAI standardized sampling methodology.

#### Selection of medicines

The WHO/HAI methodology recommends sampling of all 14 global medicines and the addition of at least 20 supplementary medicines. All medicine included in this survey were taken from the list of “clinical management of Covid-19” developed by the WHO [22] and the "national comprehensive Covid-19 management handbook" developed by the Federal Ministry of Health of Ethiopia [3]. Fourteen registered global core lists of medicines to enable international comparison and 30 supplementary medicines based on their local importance (potential to manage COVID-19 and related infection) were included in the survey (S1 Table).

### Study variables

The dependent variables of the study were availability, price, and affordability of medicines used for the management of Covid-19. The independent variables of this study were funding, selection, procurement, quantification, distribution and storage, income, and inventory management.

### Data collection tools and procedures

Three pharmacists collected the data using a standard checklist adopted from the Logistics Indicator Assessment Tool (LIAT) [23] and WHO/HAI [8]. Physical identification (observation) and abstraction of the last six months’ stock records (bin card, stock card, model 19, and model 22) of medicines in the stores and dispensaries were made to assess the availability and price of surveyed medicines. The principal investigators were the lead in the data collection process.

### Data processing and analysis

Data were initially entered and then analyzed using Microsoft Excel 2010. Medicine availability was calculated as the percent availability of individual medicines. Availability was assessed in terms of point availability and period availability. Point availability was estimated by dividing the number of medicines available during data collection divided by the total number of medicines surveyed multiplied by a hundred. Period availability was calculated by dividing the number of medicines available in the review period (6 months) divided by the total number of medicines surveyed multiplied by a hundred. Availability of medicines was defined as < 30%, 30–49%, 50–80%, and > 80% for very low, low, fairly high, and high availability, respectively [24]. Stock out of medicine is the frequency and duration of usable stock unavailability (zero on the stock records) in the store or dispensary.

The results of price and availability were analyzed for each medicine. The median Price Ratio (MPR) indicates the ratios of medicine prices relative to a standard set of international reference prices. It was determined by dividing the median local unit price by the international reference unit price. The medicine prices were obtained from the Government Employees Medical Scheme (GEMS) [25]. The MPR of medicines was not calculated for medicines where the price was not indicated in GEMS. The finding of the MPR ratio indicates how much greater or less the local medicine price is as compared to the international reference price. The local currency value (Birr) during the day of data collection was converted to dollars. Gelders S et al. criteria were used to classify the value of MPR and an acceptable local price ratio was considered to be ≤1.5 [24].

Affordability has priced the ability of the lowest-paid unskilled government workers to pay for the cost of total treatment duration. The affordability of treating health problems was estimated by multiplying the daily defined dose, median price, and the number of days of the treatment course. The daily defined dose and number of days of the treatment course were based on the standardized treatment regimens set by the Federal Ministry of Health [3]. The median prices were taken from the result of the survey. Then, the cost of treatment episodes was compared to the daily wage of the lowest-paid unskilled government worker in the town. This was allowed to determine the number of days’ wages the patient needed to pay for the full cost of treatment [26]. The daily wage of the lowest-paid unskilled government worker in the town was 0.44 Dollars (https://mywage.org/ethiopia/labour-law/wages). The formula used to calculate affordability is the total cost of medicine times thirty divided by the smallest salary of unskilled government workers [26]. The total costs of medicine for the completion of the duration of treatments for each disease were calculated. The total cost of treatment for acute health conditions was calculated by multiplying the median treatment price by the number of days required to treat the acute disease condition. The cost of medicines less than a day wage was taken as affordable, and medicines cost greater than or equal to a day wage were unaffordable. The difference in price and affordability was estimated through the Mann-Whitney test.

### Ethics approval and consent to participate

This study was ethically approved by the Ethics Review Committee of the Pharmacy Department, Wollo University (CMHS 254/13). Written informed consent was obtained from all health facilities. To maintain confidentiality, coding was used and all medical records were fully anonymized.

## Results

### Period and point availability of medicines

Medicines availability was assessed on the day of data collection (point availability) and the last six months (period availability). However, some of the medicines were not managed by public and private health facilities. Captopril 25 mg tablet, Simvastatin 20 mg tablet, Chloroquine phosphate 500 mg tablet, Chloroquine phosphate 50 mg/5ml suspension, Chloroquine phosphate 50 mg/ml injection, Hydroxychloroquine 400 mg tablet, Amoxicillin-clavulanate 250mg +62.5mg/5ml suspension, Amoxicillin-clavulanate 400mg +57mg/5ml suspension, Amoxicillin-clavulanate 200mg/5ml + 28.5 mg/5ml suspension, Ceftazidime 1 gram injection, Ceftazidime 0.5 gram injection, Ceftriaxone 0.5 gram injection, Meropenem 1 gram injection, Cefepime 0.5 gram injection, and Cefepime 1 gram injection, was not managed by public health facilities.

Chloroquine phosphate 500 mg tablet, Chloroquine phosphate 250 mg tablet, Chloroquine phosphate 50 mg/5ml suspension, Chloroquine phosphate 50 mg/ml injection and Hydroxychloroquine 400 mg tablet were not managed in private health facilities. Originator brands were not available in the past six months in public health facilities. The availability of originator brands for Glibenclamide 5mg tablet, Paracetamol 500 mg tablet, Paracetamol 250 mg suppository, and Paracetamol 125 mg suppository were 25%, 25%, 16.6%, and 25% respectively. Cotrimoxazole 240mg/5ml suspension, Amoxicillin 500 mg capsule, and Diclofenac 50 mg tablet LPG were 100% available in all public health facilities. The lowest price generic of Amitriptyline 25 mg tablet, Amoxicillin 500 mg capsule, and Diclofenac 50 mg tablet were 100% available in private health facilities (Table 1).

**Table 1:** Period availability of medicines in health facilities of Dessie town, 2021.

List of medicines	Public		Private	
	OB (%)	LPG (%)	OB (%)	LPG (%)
Salbutamol 0.1 mg/dose inhaler	0	60	0	90
Atenolol 50 mg tablet	0	20	0	85
Captopril 25 mg tablet	0	0	0	10
Simvastatin 20 mg tablet	0	0	0	50
Amitriptyline 25 mg tablet	0	80	0	100
Ciprofloxacin 250 mg tablet	0	20	0	40
Cotrimoxazole 240mg/5ml suspension	0	100	0	95
Amoxicillin 500 mg capsule	0	100	0	100
Ceftriaxone 1 g/vial injection	0	90	0	95
Diazepam 5 mg tablet	0	10	0	10
Diclofenac 50 mg tablet	0	100	0	100
Paracetamol 120mg/5ml syrup	0	70	0	75
Omeprazole 20 mg capsule	0	90	0	80
Glibenclamide 5mg tablet	0	70	25	85
Azithromycin 500 mg tablet	0	10	0	70
Chloroquine phosphate 250 mg	0	30	0	0
Paracetamol 500 mg tablet	0	40	25	70
Paracetamol 250 mg suppository	0	20	16.6	55
Paracetamol 125 mg suppository	0	70	25	85
Tramadol 50 mg capsule	0	50	0	80
Tramadol 75mg injection	0	10	0	30
Azithromycin 200mg/5ml suspension	0	10	0	60
Amoxicillin-clavulanate 875mg +125mg tablet	0	10	0	70
Amoxicillin-clavulanate 500mg +125mg tablet	0	70	0	80
Amoxicillin-clavulanate 250 mg +125mg tablet	0	50	0	20
Amoxicillin-clavulanate 125mg/5ml +31.25mg/5ml suspension	0	80	0	85
Amoxicillin-clavulanate 200mg/5ml + 28.5mg/5ml suspension	0	0	0	45
Amoxicillin-clavulanate 250mg +62.5mg/5ml suspension	0	0	0	55
Amoxicillin-clavulanate 400mg +57mg/5ml 457mg/5ml suspension	0	0	0	65
Ceftazidime 1 gram injection	0	0	0	45
Vancomycin 1 gram injection	0	10	0	65
Vancomycin 0.5 gram injection	0	10	0	20
Cefepime 1 gram injection	0	0	0	65
Meropenem 1 gram injection	0	0	0	15
Epinephrine 0.1% in 1ml ampoule injection	0	70	0	20
Dopamine 40 mg/ml injection	0	10	0	20
Hydrocortisone 50 mg/ml injection	0	20	0	80
Ceftriaxone 0.5 gram injection	0	0	0	5

**Fig 1:**
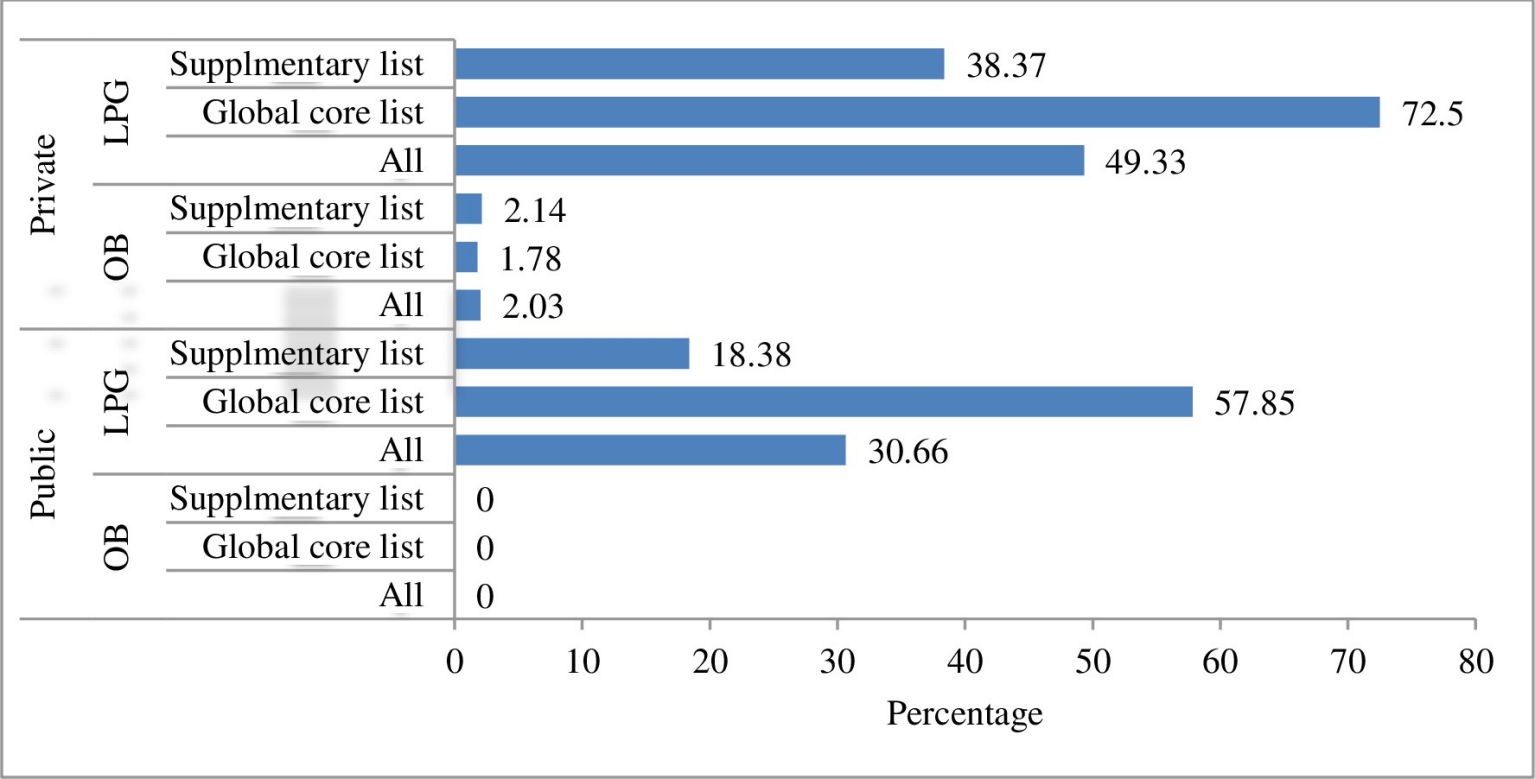
Period availability of OB and LPG medicines in health facilities of Dessie town, 2021.

The availability of OB supplementary and global core list of medicines was 0%. The availability of LPG on the global core list of medicines in private and public health facilities was 72.5 and 57.85%, respectively. The overall OB and LPG availability in private health facilities was 2.03% and 49.33%, respectively. In the public sector, the availability of OB and LPG was 0% and 30.66%, respectively (Fig 1).

During the day of data collection, 8 and 1 LPG medicines were out of stock in public and private health facilities, respectively. Amoxicillin 500 mg capsule, Omeprazole 20 mg capsule, and Diclofenac 50 mg tablet LPG were 100% available in all public health facilities. The lowest price generic of Amitriptyline 25 mg tablet, Amoxicillin 500 mg capsule, and Diclofenac 50 mg tablet were 100% available in private health facilities. None of the OB was available in public health facilities. However, only OB of Glibenclamide 5mg tablet (25%), Paracetamol 500 mg tablet (25%), Paracetamol 250 mg suppository (16.6%), and Paracetamol 125 mg suppository (25%) were available in private health facilities (Table 2).

**Table 2:** Point availability of medicines in health facilities of Dessie town, 2021.

List of medicines	Public		Private	
	OB (%)	LPG (%)	OB (%)	LPG (%)
Salbutamol 0.1 mg/dose inhaler	0	70	0	100
Atenolol 50 mg tablet	0	30	0	95
Captopril 25 mg tablet	0	0	0	10
Simvastatin 20 mg tablet	0	0	0	50
Amitriptyline 25 mg tablet	0	80	0	100
Ciprofloxacin 250 mg tablet	0	20	0	40
Cotrimoxazole 240mg/5ml suspension	0	100	0	95
Amoxicillin 500 mg capsule	0	100	0	100
Ceftriaxone 1 g/vial injection	0	90	0	95
Diazepam 5 mg tablet	0	20	0	20
Diclofenac 50 mg tablet	0	100	0	100
Paracetamol 120mg/5ml syrup	0	80	0	85
Omeprazole 20 mg capsule	0	100	0	90
Glibenclamide 5mg tablet	0	70	25	85
Azithromycin 500 mg tablet	0	10	0	70
Chloroquine phosphate 250 mg	0	30	0	0
Paracetamol 500 mg tablet	0	80	25	80
Paracetamol 250 mg suppository	0	20	16.6	55
Paracetamol 125 mg suppository	0	80	25	95
Tramadol 50 mg capsule	0	90	0	90
Tramadol 75mg injection	0	30	0	30
Azithromycin 200mg/5ml suspension	0	10	0	70
Amoxicillin-clavulanate 875mg +125mg tablet	0	10	0	70
Amoxicillin-clavulanate 500mg +125mg tablet	0	70	0	80
Amoxicillin-clavulanate 250 mg +125mg tablet	0	50	0	20
Amoxicillin-clavulanate 125mg/5ml +31.25mg/5ml suspension	0	80	0	85
Amoxicillin-clavulanate 200mg/5ml + 28.5mg/5ml suspension	0	0	0	45
Amoxicillin-clavulanate 250mg +62.5mg/5ml suspension	0	0	0	55
Amoxicillin-clavulanate 400mg +57mg/5ml 457mg/5ml suspension	0	0	0	65
Ceftazidime 1 gram injection	0	10	0	45
Vancomycin 1 gram injection	0	10	0	65
Vancomycin 0.5 gram injection	0	10	0	20
Cefepime 1 gram injection	0	0	0	65
Meropenem 1 gram injection	0	0	0	15
Epinephrine 0.1% in 1ml ampoule injection	0	70	0	20
Dopamine 40 mg/ml injection	0	10	0	20
Hydrocortisone 50 mg/ml injection	0	20	0	80
Ceftriaxone 0.5 gram injection	0	0	0	5

**Fig 2:**
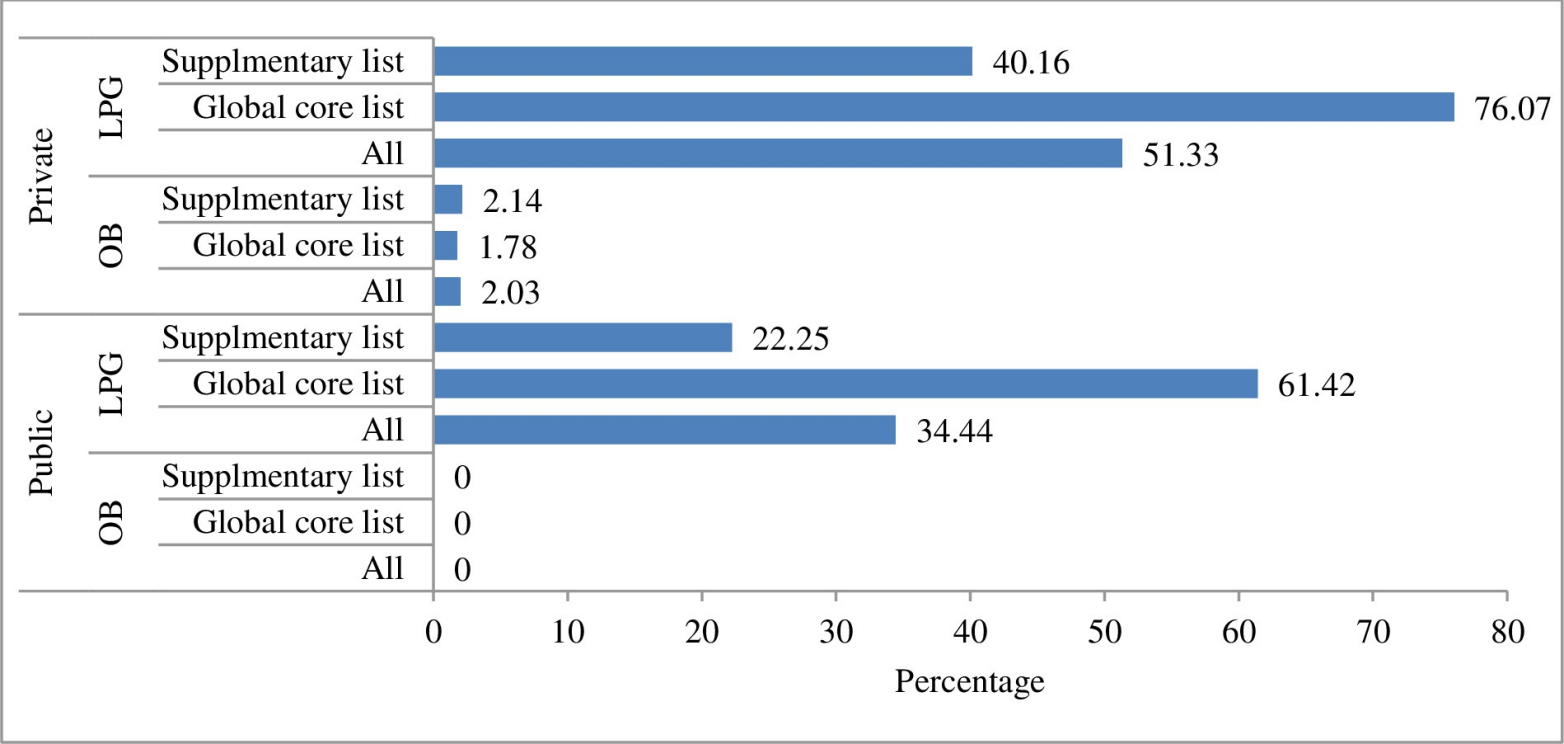
Point availability of OB and LPG medicines in health facilities of Dessie town, 2021.

The availability of supplementary LPG list of medicines was 40.16% and 22.25% in private and public health facilities of Dessie town, respectively. The availability of OB in private and public health facilities was 0% and 2.03%, respectively (Fig 2).

The frequency of public health facilities stock out medicines was ranged from 1 to 9 times. Stock out days ranged from 7 days to 295 days. On average, the number of stock out and the total number of medicines stock out days in public health facilities were 2.91 and 106.16 days, respectively. The number of stock outs in private health facilities ranged from 1 to 6 and the total number of day’s stock out ranged from 14 to 360 days. The average number of stock out and the total number of day’s stock out of medicines in public health facilities were 2.25 and 177.83 days, respectively (Table 3).

**Table 3:** Number of stock outs and the total number of day’s stock out of medicines in health facilities of Dessie town, 2021.

List of medicines	Public		Private	
	Number of stock outs	Total number of days stock out	Number of stock outs	Total number of days stock out
Salbutamol 0.1 mg/dose inhaler	2	32	1	60
Atenolol 50 mg tablet	1	7	1	14
Diazepam 5 mg tablet	5	70	5	140
Paracetamol 120mg/5ml suspension	1	120	1	240
Omeprazole 20 mg capsule	1	30	1	60
Paracetamol 500 mg tablet	9	295	6	360
Paracetamol 125 mg suppository	1	120	1	240
Tramadol 50 mg capsule	4	210	1	60
Tramadol 75mg injection	2	120	-	-
Azithromycin 200mg/5ml suspension	-	-	1	360
Amoxicillin-clavulanate 875mg +125mg tablet	4	120	4	240
Ceftazidime 1 gram injection	1	30	1	120
Cefepime 1 gram injection	4	120	4	240

### Price of medicines

The MPR of medicines in private health facilities ranged from 0.04 to 2.70. Cotrimoxazole 240mg/5ml suspension (1.80), Paracetamol 500 mg tablet (1.84), Azithromycin 200mg/5ml suspension (2.70) and Amoxicillin-clavulanate 500mg +125mg tablet (1.88) had above the acceptable MPR value (1.5) in private health facilities. Concerning public health facilities, the lowest and highest MPRs were 0.02 and 3.05, respectively. In public health facilities, Cotrimoxazole 240mg/5ml suspension (1.70), Paracetamol 500 mg tablet (1.97), Azithromycin 200mg/5ml suspension (3.05), Amoxicillin-clavulanate 500mg +125mg tablet (2.35) and Amoxicillin-clavulanate 250 mg +125mg tablet (2.08) had above 1.5 MPR value (Table 4). Of surveyed medicines, 8 (33.33%) and 6 (28.57%) had higher prices than international prices in private and public health facilities, respectively. There was no statistically significant price difference between public and private health facilities.

**Table 4:** MPR of medicines in health facilities of Dessie town, 2021.

List of medicines	Private	Public
Salbutamol 0.1 mg/dose inhaler	1.11	1.18
Atenolol 50 mg tablet	0.53	0.52
Simvastatin 20 mg tablet	1.09	NA
Amitriptyline 25 mg tablet	0.04	0.02
Ciprofloxacin 250 mg tablet	0.37	0.50
Cotrimoxazole 240mg/5ml suspension	1.80	1.70
Amoxicillin 500 mg capsule	0.81	0.60
Diclofenac 50 mg tablet	0.13	0.38
Paracetamol 120mg/5ml suspension	1.03	0.71
Omeprazole 20 mg capsule	0.06	0.40
Glibenclamide 5mg tablet	0.60	0.45
Azithromycin 500 mg tablet	0.15	0.07
Paracetamol 500 mg tablet	1.84	1.97
Tramadol 50 mg capsule	0.38	0.47
Azithromycin 200mg/5ml suspension	2.70	3.05
Amoxicillin-clavulanate 875mg +125mg tablet	0.50	0.54
Amoxicillin-clavulanate 500mg +125mg tablet	1.88	2.35
Amoxicillin-clavulanate 250 mg +125mg tablet	0.92	2.08
Amoxicillin-clavulanate 125mg/5ml +31.25mg/5ml suspension	0.68	0.42
Amoxicillin-clavulanate 250mg +62.5mg/5ml suspension	0.61	NA
Amoxicillin-clavulanate 400mg +57mg/5ml 457mg/ml suspension	1.22	NA

### Affordability of medicines

Affordability of medicines was measured in terms of the number of daily wages required to pay for treatment. Sixteen (80%) and 20 (80%) of the products were unaffordable in the public and private sectors, respectively. In the public sector, Amoxicillin-clavulanate 250 mg +125mg tablet (28.66), Amoxicillin-Clavulanate 1 gm tablet (17.24), and Cefepime 1gm vial for injection (14.63) were the most unaffordable medicines. In the public sector, drugs used for the treatment of Severe Pneumonia like Meropenem 1gm vial for injection, Ceftazidime 1gm vial for injection, Cefepime 1gm vial for injection, and Vancomycin 1gm and 0.5 mg vial for Injections required wages of 99.43, 46.93, 44.18, 21.45 and 20.84 to cover health problems, respectively (Table 5). There was no statistically significant affordability difference between public and private health facilities.

**Table 5:** Affordability of medicines in health facilities of Dessie town, 2021.

Condition	List of Medicines	Treatment schedule	Days wage to pay treatment	
			Public	Private
Antipyretics and/or analgesics	Paracetamol 500 mg tablet	1gm po every 6–8 hours, max 4gm/24 hours	0.64	0.69
Antipyretics and/or analgesics	Tramadol 50 mg capsule	50-100mg every 4–6 hours, max 400mg/24 hours	0.81	0.77
Antipyretics and/or analgesics	Tramadol 100mg/2ml ampule for Injection	50-100mg every 4–6 hours, max 400mg/24 hours	4.73	6.17
Antipyretics and/or analgesics	Paracetamol 120mg/5ml syrup	15 mg/kg every 6–8 hours	1.37	1.80
Antipyretics and/or analgesics	Paracetamol 250 mg suppository	15 mg/kg every 6–8 hours	1.38	1.18
Antipyretics and/or analgesics	Paracetamol 125 mg suppository	15 mg/kg every 6–8 hours	0.50	0.59
Immuno-modulation	Chloroquine phosphate 250 mg tablet	1gm stat, then 500mg 12 hours then 500mg BID for 5 days	0.31	0.47
Septic Shock	Epinephrine	2-30mic/min (0.1-1mic/kg/min)	2.10	1.88
Pneumonia, Mild	Azithromycin 500 mg tablet	500mg po for 3 days	1.14	2.14
Pneumonia, Mild	Amoxicillin 500mg Capsule	500 mg TID for 5–7 days	2.09	2.90
Pneumonia, Mild	Amoxicillin-Clavulanate 1 gm tablet	1 gm BID for 5–7 days	17.24	9.43
Pneumonia, Mild	Amoxicillin-Clavulanate 625mg tablet	625 mg TID for 5–7 days	6.75	12.23
Pneumonia, Mild	Azithromycin 200mg/5ml suspension	10mg/kg per day for day 1 and 5mg/kg per day for 4 days	3.08	2.01
Pneumonia, Mild	Amoxicillin-clavulanate 250 mg +125mg tablet	20-40mg/kg per day TID for 10 days	28.66	12.74
Pneumonia, Mild	Amoxicillin-clavulanate 125mg/5ml +31.25mg/5ml suspension	20-40mg/kg per day TID for 10 days	2.95	4.39
Pneumonia, Mild	Amoxicillin-clavulanate 200mg/5ml + 28.5mg/5ml suspension	20-40mg/kg per day TID for 10 days	NA	6.60
Pneumonia, Mild	Amoxicillin-clavulanate 250mg +62.5mg/5ml suspension	20-40mg/kg per day TID for 10 days	NA	6.66
Pneumonia, Mild	Amoxicillin-clavulanate 400mg +57mg/5ml 457mg/5ml suspension	20-40mg/kg per day TID for 10 days	NA	0.57
Pneumonia, Severe	Ceftazidime 1gm vial for Injection*	2gm IV TID	9.01	46.93
Pneumonia, Severe	Cefepime 1gm vial for Injection*	2gm IV TID	14.63	44.18
Pneumonia, Severe	Ceftriaxone 1gm vial for Injection*	1gm IV BID	2.74	6.91
Pneumonia, Severe	Meropenem 1gm vial for Injection*	1gm IV TID	NA	99.43
Pneumonia, Severe	Vancomycin 1gm vial for Injection*	1gm IV BID	11.75	21.45
Pneumonia, Severe	Vancomycin 0.5 gram injection*	40–60 mg/kg every 6 hours	4.25	20.84
Pneumonia, Severe	Ceftriaxone 0.5 gram injection*	75-100mg/kg every 12 hours	NA	2.46

Po: per oral, BID: twice a day, TID: three times per day, mg: milli gram, kg: killo gram, gm: gram.

* Until patients get improvement and able to take oral medication.

## Discussions

The WHO developed guideline for case management of COVID-19 in health facilities aimed to suppress viral transmission, optimizing care for patients, saving lives, and minimizing the impact of the epidemic [22]. However, there are concerns about the steady supply of drugs [2]. In this study, the availability of supplementary LPG medicines was 40.16% and 22.25% in private and public health facilities, respectively. A systematic review conducted in Ethiopia reported that the average availability of essential medicine in public and private health facilities was 70.16% and 70.1%, respectively [27]. In Eastern Ethiopia, the overall availability of LPG was 46.97% (public 42.5% and private 50.8%) [28]. Another study also reported low availability [29]. Empiric literatures in China also reported low availability of medicines in private and public health facilities [30, 31]. The availability of medicines decreased over time [32] and had a significant difference across the region of China [30]. The inadequate supply might be due to increased demand as a result of COVID-19. Pharmaceutical industries should help governments to address the unmet needs by maintaining the steady flow of pharmaceuticals. Governments should also develop strategies to balance the drug supply chain in times of crisis [5].

The present study revealed that eight and one LPG medicines were out of stock in public and private health facilities, respectively. The frequency of stock out of medicine in public health facilities varied from 1 to 9 times and ranged from 7 days to 295 days. On average, the number of stock out and the total number of days stock out of medicines in public health facilities were 2.91 and 106.16 days, respectively. In private health facilities, the number of stock outs ranged from 1 to 6 and the total number of days’ stock out ranged from 14 to 360 days. On average, medicines were stocked out 2.25 times and for 177.83 days. Ethiopian national average stock out days was 99.2 days [27]. It was also reported that less than half of the prescribed medicines were obtained from public pharmacies in Ethiopia and 17% of patients were forced to purchase medicines from private facilities [33]. The supply chain was unable to meet the growing demand for medicines used for the management of COVID-19 and made the medical supply chain to be fragile as most of the active pharmaceutical ingredients are being imported from China and India [4, 34].

The MPR of medicines ranged from 0.04 to 2.70 in private and 0.02 and 3.05 in public health facilities, respectively. Eight (33.33%) and 6(28.57%) medicines had higher prices than international prices in private and public health facilities, respectively. In public and private sectors, Cotrimoxazole 240mg/5ml suspension (1.80), Paracetamol 500 mg tablet (1.84), Azithromycin 200mg/5ml suspension (2.70) and Amoxicillin-clavulanate 500mg +125mg tablet (1.88) had above the acceptable MPR value (1.5). However, the Mann-Whitney test showed that there was no statistically significant price difference among health facilities. Findings in Ethiopia revealed that the MPR of the private sector was more than four times higher than the international purchasing price in 30% of drugs [28]. Higher pharmaceutical price was found in the United States market [35, 36]. The price difference between the public and private sectors was also apparent in China [31]. A statistically significant price difference was reported between the private and public sectors in Ethiopia [28] and China [31]. However, non-significant price variation was also reported in China [30]. It might be due to a wide price variation across countries for the same product. For example, some commonly used drugs in developing countries are more expensive than in developed countries [8]. The price of medicine is a key determinant in promoting access to medicine. The type of pharmaceutical policy used has reduced medicine procurement prices in Indonesia [37] and is recommended to be used in developing countries.

Despite 16(80%) and 20(80%) products were being unaffordable in public and private sectors respectively, it was not statistically significant. The percentage of unaffordable medicines in Ethiopia was 72.09% and 91.84% in the public and private sectors, respectively [5]. Another study reported that 63.9% of the prescribed medicines were unaffordable [38]. The price of medicines in the private sector is higher than the public sector [39]. For instance, private sourced drugs are twice expensive in Ethiopia [33]. The price of medicine matters more to poor people and poor countries as the unavailability of essential medicines turn patients to the private sector and increasing the chance of incurring catastrophic health expenditure and making treatment very difficult [27]. To increase access to medicines, affordable prices should be the main objective of any pharmaceutical system and, hence any existing barriers that hinder access to medicines should be counteractedsix [5].

The present study will help health professionals and decision-makers to improve the steady supply of medicines used for the management of COVID-19. This study was not without limitations. MPR of some medicine was not calculated as the price was not indicated in the checklist. Being a cross-sectional study, we were unable to establish a cause-and-effect relationship. Moreover, the WHO/HAI methodology allows for to assessment of availability, affordability, and price at a single point in time which made difficult to exactly understand a single point.

## Conclusions

Period and point mean availability of LPG medicines used for management of COVID-19 in private and public facilities was low and very low, respectively. In both sectors, OB availability was very low. Medicines encountered a high number of stock-outs. Fifteen and five medicines were not completely managed at all in public and private health facilities, respectively. Nearly one-third of the medicines had higher prices than international prices. Eighty percent of the products were unaffordable in both sectors. The regular supply of these medicines is crucial for better management of the disease.

## Supporting information

S1 Table List of surveyed medicines. (DOCX)

## Abbreviations

HAI: Health Action International
MPR: Median Price Ratio
WHO: World Health Organization
