# Supplementary material for: Availability, price, and affordability of medicines used for the management of Covid-19 in health facilities of Dessie town WHO/HAI survey
Source: PLoS One. 2022 Dec 21;17(12):e0279465. doi: 10.1371/journal.pone.0279465 (PMC9770383; doi:10.1371/journal.pone.0279465)
Supplement: S1 Table — (DOCX) [file pone.0279465.s001.docx]

S1 Table: List of surveyed medicines

| List of medicines | Unit |
| --- | --- |
| Salbutamol 0.1 mg/dose inhaler | Dose |
| Atenolol 50 mg tablet | Tablet |
| Captopril 25 mg tablet | Tablet |
| Simvastatin 20 mg tablet | Tablet |
| Amitriptyline 25 mg tablet | Tablet |
| Ciprofloxacin 250 mg tablet | Tablet |
| Cotrimoxazole 240mg/5ml suspension | Bottle |
| Amoxicillin 500 mg capsule | Capsule |
| Ceftriaxone 1 g/vial injection | Injection |
| Diazepam 5 mg tablet | Tablet |
| Diclofenac 50 mg tablet | Tablet |
| Paracetamol 120mg/5ml syrup | Bottle |
| Omeprazole 20 mg capsule | Capsule |
| Glibenclamide 5mg tablet | Tablet |
| Azithromycine 500 mg tablet | Tablet |
| Chloroquine phosphate 250 mg | Tablet |
| Paracetamol 500 mg tablet | Tablet |
| Paracetamol 250 mg suppository | Suppository |
| Paracetamol 125 mg suppository | Suppository |
| Tramadol 50 mg capsule | Capsule |
| Tramadol 75mg injection | Vial |
| Azithromycine 200mg/5ml suspension | Bottle |
| Amoxicillin-clavulanate 875mg +125mg tablet | Tablet |
| Amoxicillin-clavulanate 500mg +125mg tablet | Tablet |
| Amoxicillin-clavulanate 250 mg +125mg tablet | Tablet |
| Amoxicillin-clavulanate 125mg/5ml +31.25mg/5ml suspension | Bottle |
| Amoxicillin-clavulanate 200mg/5ml + 28.5mg/5ml suspension | Bottle |
| Amoxicillin-clavulanate 250mg +62.5mg/5ml suspension | Bottle |
| Amoxicillin-clavulanate 400mg +57mg/5ml 457mg/5ml suspension | Bottle |
| Ceftazidime 1 gram injection | Vial |
| Vancomycin 1 gram injection | Vial |
| Vancomycin 0.5 gram injection | Vial |
| Cefepime 1 gram injection | Vial |
| Meropenem 1 gram injection | Vial |
| Epinephrine 0.1% in 1ml ampoule injection | Ampule |
| Dopamine 40 mg/ml injection | Vial |
| Hydrocortisone 50 mg/ml injection | Vial |
| Ceftriaxone 0.5 gram injection | Vial |
| Ceftazidime 0.5 gm injection | Vial |
| Cefepime 0.5 gm injection | Vial |
| Chloroquine phosphate 500 mg tablet | Tablet |
| Chloroquine phosphate 50 mg/5ml injection | Ampule |
| Chloroquine phosphate 50 mg/ml injection | Ampule |
| Hydroxychloroquine 400 mg tablet | Tablet |
